# *Myo*-Inositol and Its Derivatives: Their Emerging Role in the Treatment of Human Diseases

**DOI:** 10.3389/fphar.2019.01172

**Published:** 2019-10-11

**Authors:** Dhani Raj Chhetri

**Affiliations:** Department of Botany, School of Life Sciences, Sikkim University, Gangtok, India

**Keywords:** *myo*-inositol, phospholipid, cyclitol, phytic acid, lipodystrophy, D-*chiro*-inositol

## Abstract

*Myo*-inositol has been established as an important growth-promoting factor of mammalian cells and animals. The role of *myo*-inositol as a lipotropic factor has been proven, in addition to its involvement as co-factors of enzymes and as messenger molecules in signal transduction. *Myo*-inositol deficiency leads to intestinal lipodystrophy in animals and “inositol-less death” in some fungi. Of late, diverse uses of *myo*-inositol and its derivatives have been discovered in medicinal research. These compounds are used in the treatment of a variety of ailments from diabetes to cancer, and continued research in this direction promises a new future in therapeutics. In different diseases, inositols implement different strategies for therapeutic actions such as tissue specific increase or decrease in inositol products, production of inositol phosphoglycans (IPGs), conversion of *myo*-inositol (MI) to D-*chiro*-inositol (DCI), modulation of signal transduction, regulation of reactive oxygen species (ROS) production, etc. Though inositol pharmacology is a relatively lesser-known field, recent years of research has generated a critical mass of information on the subject. This review aims to summarize our current understanding on the role of inositol derivatives in ameliorating the symptoms of different diseases.

## Introduction

Inositols are polyols having six-carbon ring structure where each carbon is hydroxylated. A number of these sugar-alcohol isomers are biologically active, of which *myo*-inositol (MI) is the most common (Majumdar et al., 1997). It constitutes a component of membrane phospholipids and mediates osmoregulation (Majumder and Biswas, 2006). Its phosphorylated derivatives act as second messengers in signal transduction pathways (Berridge, 2009), mediate phosphorylation of proteins (Saiardi et al., 2004), participate in chromatin remodeling and gene expression (Odom et al., 2000; Shen et al., 2003), and facilitate mRNA export from the nucleus (York et al., 1999).

Altered MI levels have been observed in the brains of patients of Alzheimer’s disease, those suffering from mental disorders, and suicide and stroke victims (McLaurin et al., 1998; Macri et al., 2006). High fetal inositol concentrations in the cerebrospinal fluid have been attributed to the pathogenesis of Down’s syndrome (Acevedo et al., 1997). Administration of MI has been found to be therapeutic for obsessive-compulsive disorder and panic disorder (Seelan et al., 2009). Lower frontal cortex MI is linked to the pathophysiology of depression and concomitant sleep symptoms (Urrila et al., 2017).

MI deficiency causes high accumulation of triacylglycerol, cholesterol, and non-esterified lipids in the mammalian liver. A minimum threshold level of free MI deters the formation of fatty liver (Burton and Wells, 1974; Hayashi et al., 1974a; Hayashi et al., 1974b). Hence, the metabolic understanding of MI status in any biological organ or system is primarily dependent on MIPS activity and its regulation.

### Chemistry

**Figure 1:**
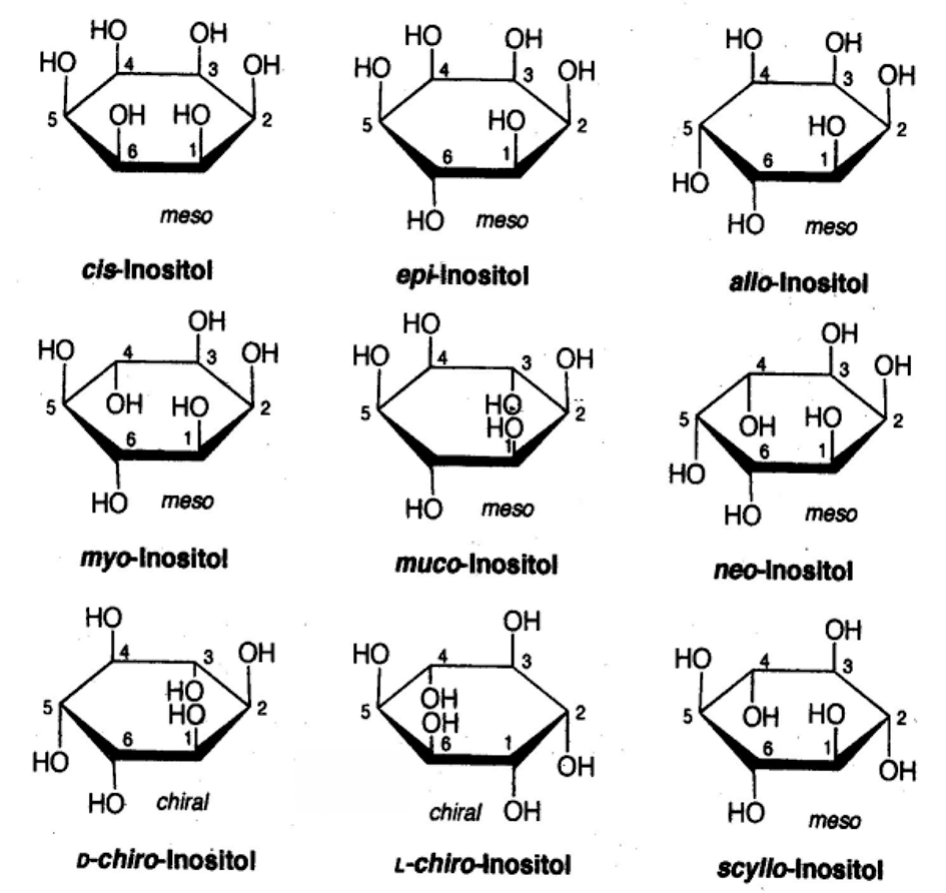
The planar structure of nine possible stereoisomers of inositol.

Chemically inositols are isomers of hexahydroxy-cyclohexanes. Among the nine possible geometrical isomers of inositol, seven are optically inactive or “meso,” and the remaining two form a chiral pair. The planar structures of the different isomers of this compound are presented in **Figure 1**. The molecule of MI has one axial and five equatorial hydroxyl groups. The axial hydroxyl group at position 2 is most stable to hydrolysis.

L-*myo*-inositol-1-phosphate synthase (MIPS) catalyzes the first step in the biosynthesis of all *myo*-inositol-containing compounds (Seelan et al., 2009). It converts glucose-6-phosphate to *myo*-inositol-1-phosphate (MIP). The phosphate moiety in MIP is subsequently removed by *myo*-inositol-1-phosphatase (IMPase) to produce free MI (Majumder and Biswas, 2006).

In addition, this compound could also be produced by cyclic synthesis (Agranoff et al., 1958; Paulus and Kennedy, 1960) and the hydrolysis of phosphatidylinositol. Although MIP is the intermediate common to both pathways, two different forms of compound are produced, the L-form by the synthetic pathway and the D-form by the cyclic pathway (Parthasarathy and Eisenberg, 1986). However, both the isomers are catalyzed by IMPase (Eisesnberg, 1967), which produces MI.

### *Myo*-Inositol in Disease and Medicine

Elevated MI levels have been observed in Alzheimer’s disease, gliomatosis cerebri, diabetes mellitus, systemic lupus erythematosus, multiple sclerosis, etc. Further, decreased brain levels of MI were observed in chronic hepatic and hypoxic encephalopathy, stroke, acute thyrotoxic Graves’ disease, toxoplasmosis, cryptococcosis, and lymphoma (Haris et al., 2011). In the following section, important diseases that are influenced by MI and its derivatives (**Table 1**) have been reviewed.

**Table 1:** Pharmacological effects of myo-inositol and its derivatives against different disease symptoms.

Effective inositol derivative	Affected tissue/organ	Disease/symptom	Reference
MI	Intestine (gerbil)	Lypodystrophy	Hegsted et al., 1973
MI	Heart (rat)	Left ventricular stiffness	Regan et al., 1973
MI	Liver (rat)	Fatty liver disease	Burton and Wells, 1974; Hayashi et al., 1974a; Hayashi et al., 1974b
MI	Brain (human)	Affective disorder	Barkai et al., 1978
MI	Lung (mouse)	Tumor	Wattenberg, 1996
MI	Brain (human)	Suicidal tendency	Shimon et al., 1997
MI	Liver (mouse)	Cancer	Nishino et al., 1999
MI	Lung (human)	Tumor	Jyonouchi et al., 1999
IP_6_	Heart (rat)	Calcification of vessels	Grases et al., 2000
DCI-IPGs	Ovary (human)	PCOS	Sabuncu et al., 2001
DCI	Diabetic human	Endothelial dysfunction	Larner, 2002
DCI	Diabetic human	Endothelial dysfunction, metabolic syndrome, erectile dysfunction	Nacimento et al., 2006
PIP_3_	Nerve tissue, thyroid gland, colon, lung, prostate gland, skin (human)	Cancer	Luo et al., 2003; Osaki et al., 2004; Yuan and Cantley, 2008
IP_6_ + MI	Colon, breast, lung (human)	Cancer	Vucenic and Shamsuddin, 2003; Vucenic and Shamsuddin, 2006
MI	Brain (human)	Depression	Coupland et al., 2005; Shirayama et al., 2017
MI	Fetal brain (human)	Down’s syndrome	Seelan et al., 2009
PIP_2_	Ovary, breast, lung, colon, stomach (human)	Cancer	Engelman et al., 2006; Yuan and Cantley, 2008
MI	Brain (human)	Bipolar disorder	Saltiel et al., 2007
MI	Brain (human)	Mild cognitive disorder	Berman et al., 2008; Voronov et al., 2008
MI	Brian (human)	Alzheimer’s disease	Siger et al., 2009
DCI/MI ratio	Diabetic human	Insulin resistance	Larner et al., 2010
MI	Lung (mouse)	Tumor	Kassie et al., 2010
IP_6_ + MI	Breast (human)	Cancer	Bacic et al., 2010
IP_6_	Prostate gland (human)	Cancer	Gu et al., 2010
DCI	Ovary (human)	Poor oocyte quality	Carlomagno et al., 2011; Isabella and Raffone, 2012; Simi et al., 2017
PIP_2_	Brain (human)	Schizophrenia	Lo Vasco et al., 2012
MI + DCI	Ovary (human)	PCOS	Minozzi et al., 2013

DCI, D-chiro-inositol; ED, endothelial dysfunction; IP_6_, Myo-inositol hexakisphosphate/phytic acid; IPG, inositol phosphoglycan; MI, Myo-inositol; PIP_2_, phosphatidylinositol inositol 4,5-bisphosphate; PIP_3_, phosphatidyl inositol 3,4,5-trisphosphate.

### Dyslipidemia and Cardiac Diseases

Ever since it was known that MI deprivation in diet resulted in fatty liver condition in rats (Burton and Wells, 1974, Hayashi et al., 1974a; Hayashi et al., 1974b), the interest regarding its therapeutic value developed. MI also reduced the accumulation of hepatic triglyceride in the liver (McCrea and Camilli, 2009). Treatment with MI assisted in the removal of cholesterol from the myocardium, resulting in the decrease in lipid buildup in the heart that improved heart function. The reduction in myocardial lipid content ultimately resulted in the decrease in left ventricular stiffness (Regan et al., 1973).

A family of proteins called myotubularins which are actually inositol-3-phosphatases that dephosphorylate PI_3_P and PI(3,5)P_2_ are implicated in cardiomyopathy. It has been found that mutations in the genes coding for the aforementioned proteins caused cardiomyopathy (McCrea and Camilli, 2009). Nebivolol, a beta-blocker drug, induces vasorelaxation through activation of inositol phosphate metabolism (Parenti et al., 2000). Calcification of heart vessels is an undesirable attribute of cardiovascular disease (CVD), and IP_6_ acts as a crystallization inhibitor of calcium salts *in vitro*, reducing the calcification of coronary arteries (Grases et al., 2000).

### Diabetic Complications

It is a known fact that, in diabetic animals, there is limited metabolism of fructose in the nerve system leading to the accumulation of sorbitol and fructose, which is responsible for peripheral neuropathy (Grabby, 1973). This causes a decrease in the motor nerve conduction velocity as well as in the MI concentration of the sciatic nerve at the onset of diabetes (Greene et al., 1975). These anomalies could be prevented by the exogenous administration of MI (Palmano et al., 1977).

Endothelial dysfunction (ED) caused by hyperglycemia and hyperlipidemia is an early feature of diabetes (Nacimento et al., 2006). Inositol phosphoglycans (IPGs) are generated rapidly in response to insulin and have an insulin-like effect *in vivo* and *in vitro* (Huang et al., 1999). In human urine, the level of *chiro*-inositol is decreased, while the MI content increased in diabetic subjects. The decreased urinary *chiro*-inositol is inversely correlated to insulin resistance. Administration of D-*chiro*-inositol (DCI) in diabetic humans effectively decreased hyperglycemia and hypertriglyceridemia (Larner, 2002).

In type 2 diabetic subjects, the higher levels of MI and the lower levels of DCI are referred to as inositol imbalance. *Chiro-*inositol deficiency and imbalance with *myo*-inositol are directly related to insulin resistance (Larner et al., 2010).

### Cancer

Carcinogenesis in various organs may be inhibited by MI. Significant suppression of liver carcinogenesis by the oral administration of MI has been observed in mice (Nishino et al., 1999). Benzo[a]pyrene (B[a]P), a carcinogen derived from tobacco, causes lung tumor in rodents through its metabolite, anti-7,8-dihydroxy-9,10-epoxy-7,8,9,10-tetrahydrobenzo[a]pyrene (B[a]PDE). Interestingly, the same metabolite inhibits the differentiation of small airway epithelial cells (SAE) in humans. MI protects SAE cells against such inhibitory effects (Jyonouchi et al., 1999). When MI was added to dexamethasone (another compound that prevents pulmonary neoplasia), an additive effect was observed on the inhibition of lung carcinogenesis (Wattenberg, 1999). Administration of MI decreases the multiplicity and size of surface tumors. It also decreases the size of adenocarcinoma, and therefore, it may be utilized for the chemoprevention of early pulmonary lesions (Kassie et al., 2010).

Striking anticancer effects of IP_6_ and inositol have been demonstrated in experimental models (Vucenic and Shamsuddin, 2003). In colon, breast, and metastatic lung cancer models, the effect of the combination of IP_6_ and MI was significantly better than by either of the two acting alone (Vucenic and Shamsuddin, 2003, 2006). IP_6_ also inhibits prostate cancer (Pca) cell proliferation and stimulates their apoptotic death. IP_6_ inhibits constitutive and growth factor-induced signaling pathways, which eventually leads to the inhibition of growth and the induction of apoptotic death of Pca cells (Gu et al., 2010).

### Mental Afflictions and Cognitive Diseases

Evidences suggest that the MI level in brain is associated with changes in mood state. The MI levels in the frontal coretex of suicide victims and those suffering from bipolar disorder were 23 and 30% lower, respectively, than the normal levels (Shimon et al., 1997). Patients of major depressive disorder have shown significantly lower MI/creatine ratios. The low levels of MI in the prefronatal/anterior cingulate cortex in major depressive disorder patients may be a consequence of altered glial metabolism (Coupland et al., 2005). Abnormal level of MI along with glutamate and glutamine was found in the brains of major depressive patients (Shirayama et al., 2017).

Administration of lithium causes a lowering of MI in the critical areas of the brain, and the effect is therapeutic. Lithium reduces MI level in the right frontal lobe in the brains of patients with manic depression. On the other hand, valproic acid (VPA) decreases the intercellular concentrations of inositol by inhibiting the key enzyme of MI biosynthesis, MIPS, in the human brain (Saltiel et al., 2004). Derivatives of VPA, valnoctamide (VCD), and valrocemide (VGD) are potent anticonvulsant drugs (Loscher and Nau, 1985; Anderson et al., 1992). The fact that 1mM VCD and VGD drastically inhibited human brain MIPS activity supports the view that these derivatives act as potential mood stabilizers (Saltiel et al., 2007).

The extra chromosome 21 in Down’s syndrome (DS), which leads to dementia later in life, is phenotypically similar to Alzheimer’s disease (AD). The presence of approximately 50% higher level of MI in DS patients suggests a gene dose effect of the extra chromosome 21, where the human osmoregulatory sodium/*myo*-inositol cotransporter gene is located. Still, higher levels of MI in older adults with DS are similar to that symptomatic of AD (Huang et al., 1999).

Synaptojanin-1 is a polyphosphosphoinositide phosphatase found in the neurons may have a role in the early onset of AD associated with DS. This enzyme is responsible for maximum of the PIP_2_ phosphatase activity in the brain and plays a critical role in synaptic transmission (Di Paolo and DeCamilli, 2006). The AD peptide Aβ42 stimulates PIP_2_ cleavage and leads to abnormal PIP_2_ metabolism in AD (Berman et al., 2008). The genes encoding synaptojanin-1 as well as the Aβ42 precursor is located in chromosome 21, the triplication of which is responsible for DS (McCrea and Camilli, 2009). In DS patients, the level of synaptojanin-1 is increased, and the corresponding level of PIP_2_ is decreased.

People with mild cognitive disorder (MCI) have higher risk of conversion to AD. In MCI, increased manifestation of MI occurs in the parietal white matter (WM), while in AD, the elevation of MI was found throughout the WM (Zhu et al., 2006). Therefore, MI level in MCI may be regarded as an early indicator of AD (Siger et al., 2009).

Abnormalities in signal transduction play a role in the development of mood disorders. Activated PI-PLC cleaves PIP_2_ into IP_3_ and DAG, both of which are crucial molecules for signal transduction (Suh et al., 2008). Different PI-PLC enzymes are tissue-specific, and the different expression of some isoforms was described in pathological conditions (Lo Vasco et al., 2013). A role of PI-PLC β1 in mood disorders has been suggested (Lo Vasco et al., 2012), and this hypothesis is in sync with the data obtained from schizophrenia models (Mc Omish et al., 2008). PI-PLC β1 was also suggested to represent a molecular convergence point of several neurotransmitter pathways implicated in schizophrenia (Choi et al., 1989; Kim et al., 1997).

### Polycystic Ovary Syndrome

Polycystic ovary syndrome (PCOS) is the most common form of the endocrine metabolic diseases affecting 6–10% of women of reproductive age (Diamanti-Kandarakis et al., 1999). Insulin resistance (IR) and compensatory hyperinsulinemia play an integral role in the pathogenesis of this syndrome (Nestler, 1997). IR places these women at an increased risk of the development of cancer, hypertension, dyslipidemia, type 2 diabetes, and CVDs (Burghen et al., 1980). It is known that some functions of insulin require low molecular weight IPGs (Dona et al., 2012) and also that a deficiency in DCI containing IPGs and/or altered DCI metabolism may contribute to IR.

Besides IR, hyperandrogenism is another feature of PCOS. This hyperandrogenism is related to alteration of steroidogenesis in ovary and adrenal glands (Reyes-Munoz et al., 2018). Androgens act synergistically with follicle stimulating hormone (FSH) and modify steroidogenesis enzymes (Lenie and Smitz, 2009) which is also related to IR.

In women with PCOS, administration of DCI improves clinical features of the syndrome (Baillargeon et al., 2010). Moreover, combined therapy of MI and DCI improves the metabolic profile of obese PCOS patients, reducing the risk of CVD (Minozzi et al., 2013). MI may be incorporated into membrane phosphatidylinositols, or it may constitute IPGs in response to insulin. After its release, the IPGs interact with tissues involved in insulin action, thus potentiating the effects of insulin (Cheang et al., 2008). PCOS patients also exhibit an increased DCI/MI ratio (i.e., overproduction of DCI). This in turn leads to MI deficiency in the ovary. A balance between the two inositols is associated with IR and sensitivity (Heimark et al., 2013).

### Epilepsy

In case of patients with temporal lobe epilepsy (TLE), MI level increases in the areas of seizure focus (temporal lobe) and its concentration decrease in the areas of seizure spread i.e., frontal lobe (Wellard et al., 2003). In the temporal lobe, the increased MI has been reported as a consequence of induction of Na+/MI cotransporter1 (SMIT1) after seizure activity in the area of seizure focus (Nonaka et al., 1999). The decreased MI in the frontal lobe reflects the osmolyte changes due to secondary effect of seizures. MI is transported from extracellular fluid into the cell through SMIT1. Overexpression of SMIT1 as well as MI supplementation increases intracellular phosphoinositide level and thereby alters phosphoinositide modulated ion channels suggesting the role of SMIT1 in signaling (Dai et al., 2016).

In experimental rats, MI treatment significantly reduces the severity of status *epilepticus* induced by kianic acid. The treatment reduced both the frequency and duration of spontaneous recurrent seizures, the main character of epilepsy. In addition, MI had significant effects on SMIT1 and leucine rich repeat-containing 8A, a component of volume regulated anionic channel (Tsverava et al., 2019).

The IMPA2 gene located at human chromosome 18p 11.2 is responsible for febrile seizure (FS). IMPA2 codes for myo-inositol monophosphatase 2 that converts inositol monophosphate to MI and plays important role in phosphatidylinositol signaling pathway (Nakayama et al., 2004).

## Discussion

It is a foregone conclusion that MI and its derivatives exert various metabolic actions generating therapeutic outcomes. The activities are due to reduction in ROS generation, direct superoxide scavenging, protection of NO signaling, etc. For example, DCI may be considered a therapeutic agent against metabolic syndrome, endothelial dysfunction, and erectile dysfunction in diabetes patients (Nacimento et al., 2006), and MI may act as alternative of metformin, the most popular oral antidiabetic drug, because it interacts directly with insulin target tissues; however, it does not show the side effects of the drug (Dona et al., 2012). The insulin like action of MI and DCI is due to the production of inositol glycan secondary messengers. These inositol glycans may modulate cell signaling, and in addition, inositols are incorporated in cell membrane phospholipids (Lagana et al., 2018).

Depression and schizophrenia are severe psychiatric diseases that affect millions of individuals worldwide, consequently increasing global suicide levels (Ren, 2019). MI and its derivatives may be a very important adjunct therapy in such cases. The most important role of MI is found in the treatment of bipolar disorder. More often than not, lithium is the first line of defense in such cases. However, lithium treatment often leads to psoriasis and depression. In such cases, MI may act as a preferable alternative since it is effective in mood stabilization as well as in the treatment of psoriasis (Kontoangelos et al., 2010).

The importance of inositol in cancer lies in the fact that inositol-3-phosphatase is a potent tumor suppressor, and its mutation leads to many types of cancers (Luo et al., 2003; Osaki et al., 2004; Yuan and Cantley, 2008). On the other hand, activating mutations in PI_3_-kinases have been reported in ovarian, breast, lung, colon, and gastric cancers (Engelman et al., 2006; Yuan and Cantley, 2008). In addition, in breast cancer patients, IP_6_ and MI may be a valuable adjunctive therapy. They also help in ameliorating the side effects and improving the quality of life (Bacic et al., 2010).

In oligoasthenospermia (OA), reduction in the number and motility of spermatozoa takes place. MI plays a crucial role in the osmoregulation of seminal fluid, thereby improving sperm motility. The antioxidant effect of MI also plays important role in the production and regulation of spermatozoa. Therefore, MI could be used in OA patients undergoing an *in vitro* fertilization cycle (Gulino et al., 2016). ROS affects not only the morphology and motility of spermatozoa but may also damage mitochondrial membrane potential (MMP) which in turn increases ROS production. MI may improve the sperm mitochondrial function, thereby improving sperm parameters in OA patients (Condorelli et al., 2017).

In women with PCOS, the combined therapy of MI plus DCI is able to influence the metabolism leading to improved lipid profile. However, in these patients, enhanced epimerization of MI to DCI takes place in the ovary, leading to excess DCI and less MI (Isabella and Raffone, 2012). This MI depletion eventually leads to poor oocyte quality (Carlomagno et al., 2011). MI supplementation may salvage the situation by improving oocyte quality (Simi et al., 2017).

The spurt of research on inositol biochemistry started from the 1960s with different groups taking the lead on various aspects of the work (Ballou and Pizer, 1960; Shaktin and Tatum, 1961; Nagai and Funahashi, 1962, Plouvier, 1963; Michell and Hawthorne, 1965; Charalampous and Chen, 1966; Eisesnberg, 1967; Sherman et al., 1968; Dittmer and Douglas, 1969). Now, the molecules have again come into focus primarily due to the continued increase in lifestyle diseases and the long quest for effective and non-toxic cure for the same. ROS reduction may be one of the strategies of MI derivatives for its therapeutic functions. In diabetes and heart ailments, ROS generation by NADPH oxidase action and mitochondrial disruption could be inhibited by DCI (Vendrov et al., 2015). IP_3_ signaling may be another mechanism by which MI derivatives influence cellular functions. IP_3_ is responsible for Ca^2+^ release from the ER which raises the cytosolic Ca^2+^, and this in turn activates many enzymes and proteins. Indeed, defective IP_3_ receptors have been found responsible for many neurodegenerative disorders (Egorova and Bezprozvanny, 2018). Thus, MI and its derivatives may well play important roles not only to ameliorate cancers and psychotic diseases but also in many lifestyle diseases like obesity, diabetes, CVD, etc. This report tried to highlight these critical areas.

## Author Contributions

DC conceptualized the article, prepared the manuscript and did the editing.

## Funding

Supported by National Mission on Himalayan Studies (NMHS) grant of the Government of India (Ref. No. NMHS/MG-2016/005).

## Conflict of Interest

The author declares that the research was conducted in the absence of any commercial or financial relationships that could be construed as a potential conflict of interest.
